# Identification of Immune Cell Infiltration Landscape and Their Prognostic Significance in Uveal Melanoma

**DOI:** 10.3389/fcell.2021.713569

**Published:** 2021-08-26

**Authors:** Han Zhao, Yun Chen, Peijun Shen, Lan Gong

**Affiliations:** ^1^Department of Ophthalmology, Eye, Ear, Nose, and Throat Hospital of Fudan University, Shanghai, China; ^2^Laboratory of Myopia, NHC Key Laboratory of Myopia, Chinese of Medical Sciences, Fudan University, Shanghai, China; ^3^Shanghai Key Laboratory of Visual Impairment and Restoration, Fudan University, Shanghai, China; ^4^Department of Stomatology, The Second Xiangya Hospital, Central South University, Changsha, China; ^5^Department of Gastroenterology, The Third Xiangya Hospital of Central South University, Changsha, China; ^6^Hunan Key Laboratory of Non-resolving Inflammation and Cancer, Central South University, Changsha, China

**Keywords:** uveal melanoma, prognosis, immune cell infiltration, ICI scores, immunotherapy, tumor microenvironment

## Abstract

Uveal melanoma (UVM) is the most common primary intraocular cancer in adults. Increasing evidence has demonstrated that immune cell infiltration (ICI) is crucial in predicting patient outcomes and therapeutic efficacy. Thus, describing the immune cell infiltrative landscape of UVM tumors may yield a novel prognostic marker and provide direction for immunotherapeutic selection. In this study, the gene expression data and clinical information of UVM patients were obtained from the cancer genome atlas (TCGA) and gene expression omnibus (GEO) databases. The ICI landscape of UVM was analyzed using the CIBERSORT and ESTIMATE algorithms. Two ICI phenotypes were defined, and the ICI scores were calculated by using principal component analysis algorithms. We found that a subtype with high ICI scores had poorer prognosis and increased expression levels of immune checkpoint-related genes. This study demonstrates that ICI scores are an independent prognostic biomarker and highlights their value in predicting immunotherapeutic outcomes.

## Introduction

Uveal melanoma (UVM) is an aggressive primary intraocular cancer that originates from melanocytes in the eye. Within the past few decades, the incidence of UVM has remained stable (Virgili et al., 2007). The mean age-adjusted incidence is 5.1 per million in the United States (Singh et al., 2011). UVM has a strong propensity to metastasize from the eye to other organs. Despite the significant improvements in treatment, including enucleation, resection, and radiation therapies, up to 50% of UVM patients will eventually develop metastatic disease, with many cases suffering from fatal liver metastasis (Eskelin et al., 2000; Singh et al., 2011). Extensive research has shown that UVM patients develop micro-metastases early after initial diagnosis, and the median survival of patients with metastatic progression ranges from 4 to 15 months (Eskelin et al., 2000; Kujala et al., 2003). Unfortunately, existing therapies are insufficient to treat distant metastases (Augsburger et al., 2009). Over the past several decades, targeted therapies and immunotherapy, such as immune checkpoint blockade (ICB), vaccination, and adoptive T-cell therapy, have been proven efficacious in multiple types of cancers (Curran et al., 2010; Seiwert et al., 2016). However, one major limitation of immunotherapy is that a clinical response is observed in only approximately 0 to 5% of patients (Heppt et al., 2017). Thus, it is crucial to identify novel therapeutic markers of immunosuppression in UVM.

The tumor microenvironment (TME) plays a crucial role in melanoma initiation, evolution, metastasis, and relapse. The TME in UVM contains numerous non-tumor cells and stromal cellular elements, including immune cells, inflammatory cells, endothelial cells, and mesenchymal cells (Hanahan and Coussens, 2012). For instance, proangiogenic tumor-associated macrophages (TAMs) can facilitate the homing, extravasation, and metastases to the liver in UVM (Van den Eynden et al., 2013). The density of tumor-infiltrating lymphocytes (TILs) is closely correlated with the development of metastatic UVM and predicts poor prognosis (Singh et al., 2001). TILs and the cytokines they produce dampen natural killer (NK) cell effector function (Javed and Milhem, 2020). TILs play an essential role in the response to ICB (Rosenberg et al., 2016). However, unlike cutaneous melanoma, only about 10% of primary UVM tumors express anti-programmed death-ligand 1 (PD-L1) (Kaunitz et al., 2017), though up to 50% of TILs express the receptor, PD-1 (Javed et al., 2017). However, the association between TME components and the effectiveness of immunotherapy has not been fully elucidated.

Studies have shown that genetic or epigenetic alterations are involved in the tumorigenesis and progression of UVM and can affect the TME (Li et al., 2018; Fallico et al., 2021). The frequencies of oncogenic *BRAF* and *NRAS* mutations in UVM are approximately 40 to 60% and 15 to 20%, respectively (Flaherty et al., 2010; Mandalà et al., 2014). Loss of one copy of a chromosome (monosomy 3) or BAP1 deficiency is also involved in the progression of metastatic UVM, while chromosome 8 alterations are associated with poor prognosis (Thomas et al., 2012). In addition, *GNAQ* and *GNA11* mutations are associated with melanoma cell proliferation and metastasis (Huang et al., 2015). Aberrant DNA methylation of genes contributes to metastatic progression and poor survival (Ness et al., 2021). Moreover, non-coding RNAs (ncRNAs) have also been investigated in UM (Bande et al., 2020). The ncRNAs including microRNAs, long non-coding RNAs, and circular RNA regulate gene expression, playing important roles in UVM development and progression (Yang et al., 2018). Falzone et al. (2019) reported that a set of miRNAs could be used as biomarkers in UVM. Recent studies found that a higher tumor mutational burden is associated with elevated responses to ICB compared with tumors with a lower mutation burden (Yarchoan et al., 2017).

In the present study, we explored the intratumoral immune infiltration landscape in UVM using the cancer genome atlas (TCGA) and gene expression omnibus (GEO) databases with the CIBERSORT and ESTIMATE algorithms. We used immune cell infiltration (ICI) scores to characterize the immune cell landscape and to estimate the prognosis of UVM patients. Moreover, we also estimated the TMB pattern in patients from low- and high-ICI score groups. Previous studies have shown that gender and age are associated with the immunotherapy response and prognostic outcomes across different cancer types (Sceneay et al., 2019; Ye et al., 2020). Thus, we also investigated the feasibility of applying ICI scores to different genders and age groups in UVM.

## Materials and Methods

### Data Collection From the TCGA and GEO Databases

A total of 80 UVM sample datasets from the TCGA database were downloaded via The University of California Santa Cruz (UCSC) Xena browser^1^, including gene expression profiles [fragments per kilobase million (FPKM) value], their clinical data, and mutation data. The gene expression profiles of the TCGA-UVM dataset (FPKM value) were then transformed into transcripts per kilobase million (TPM), which was more closely aligned with the microarray data. We selected GEO datasets with the following criteria: (1) the sample size in the dataset was more than 60; (2) the datasets contained gene expression profiling data; (3) the datasets contained patients’ clinical and prognostic data. Finally, GSE22138 with 63 samples was chosen (platform GPL570, Affymetrix Human Genome U133 Plus 2.0 Array). Strawberry Perl (version 5.32.0^2^) was used to extract the gene expression data from the TCGA-UVM and GSE22138 datasets and construct a data matrix for further analysis. The clinical and pathological characteristics of each patient in the TCGA-UVM cohort and GSE22138 are summarized in Supplementary Table 1.

### Immune Cell Infiltration Analysis

Immune cell infiltration levels in UVM tumors were estimated with the CIBERSORT (Cell-type Identification by Estimating Relative Subsets of RNA Transcripts) algorithm (Chen et al., 2018). Based on a set of reference gene expression data (LM22), the “CIBERSORT” R package was used to analyze the relative expression levels of 22 immune cell types in individual tissue samples from the TCGA-UVM and GSE22138 databases. CIBERSORT is a deconvolution algorithm, which can infer cell-type proportions in data from tumor samples with mixed cell types by using support vector regression based on LM22 datasets. LM22 includes 22 different immune cells, including naive B cells (Bn), Bm, plasma cells, CD8^+^ T cells, naive CD4^+^ T cells (CD4^+^ Tn), CD4^+^ resting memory T cells (CD4^+^ Tmr), CD4^+^ memory-activated T cells (CD4^+^ Tma), Tfh, Tregs, γδT, resting natural killer cells (NKr), activated natural killer cells (NKa), monocytes, M0 macrophages (M0), M1 macrophages (M1), M2 macrophages (M2), resting dendritic cells (DCr), DCa, resting mast cells (Mr), activated mast cells (Ma), eosinophils, and neutrophils.

### Tumor Microenvironment Analysis

Immune and stromal contents from each UVM sample were evaluated by the “ESTIMATE” R package. Specifically, the ESTIMATE score was calculated as the sum of the immune and stromal scores, which represents the abundance of immune and stromal components, respectively. Higher ESTIMATE Scores correspond to lower tumor purity (Yoshihara et al., 2013).

### Establishment of Consensus Clustering Based on Immune Cell Infiltration

The consensus clustering (CC) method was used to estimate the number of unsupervised classes in a dataset. Based on the ICI profile, we classified patients in TCGA-UVM and GSE22138 into various ICI clusters using the “ConsensusClusterPlus” R package. The procedure was repeated 1,000 times to ensure classification stability and reproducibility, which were visualized using the heat map function in R software.

### Differentially Expressed Genes (DEGs) Screening

We classified all UVM patients into different ICI clusters based on the results of the CIBERSORT analysis. The “Limma” R package was utilized to identify DEGs among different ICI subtypes, and DEGs with an adjusted *P* value < 0.05 and |logFC| ≥ 1 were considered to show a significant difference.

### Calculation of Immune Cell Infiltrating Score

The calculation of ICI scores was performed as follows. The calculation of ICI scores was performed as follows. Based on DEGs values, an unsupervised clustering was performed to categorize the patients in TCGA-UVM and GSE22138 datasets into several groups for further analysis. According to the positive and negative relationship between the DEGs and the cluster signature, the ICI genes were divided into two groups, namely ICI gene signatures A and B. Next, the “clusterProfiler” R package was used to annotate genes. The principal component analysis (PCA) was then used to reduce the dimension of the ICI gene subgroup based on the Boruta algorithms. Each patient was then assigned an ICI score and classified into a high- or low-ICI group via its corresponding median risk score of a cohort. The ICI of each UVM sample was calculated using the formula: ICI score = Σ PCI_A_ - Σ PCI_B_.

### Gene Set Enrichment Analysis

Gene set enrichment analysis is a computational method used to determine whether an *a priori* defined set of genes shows statistically significance and concordant differences between two biological states (Subramanian et al., 2005). In this study, the Kyoto encyclopedia of genes and genomes (KEGG) gene sets (v7.4) was downloaded from the Molecular Signatures Database (MSigDB^3^). Gene set enrichment scores were calculated based on genes in low- and high-ICI scores groups using GSEA software (v4.1.0^4^). NOM *p*-value < 0.05 and FDR *q*-value < 0.25 was considered as statistically significant. The top 5 KEGG pathways were selected and visualized.

### Somatic Alteration Analysis

The mutation status of patients in the TCGA-UVM was downloaded using the TCGA Genomic Data Commons (GDC^5^). The “maftool” R package was used to determine the tumor mutational burden of TCGA-UVM and to evaluate the difference in TMB between high- and low-ICI score groups.

### Gene Expression and Clinical Data Sets With ICB Therapy

For the TCGA-SKCM cohort, the expression profiles (FPKM values) downloaded from the UCSC Xena browser were transformed into TPM values. A total of 470 skin melanoma samples were used to calculate ICI scores. For patients with advanced melanoma treated with MAGE-3 antigen-based immunotherapy (GSE35640, *n* = 55; GPL570, Affymetrix Human Genome U133 Plus 2.0 Array), gene expression and clinical data were downloaded from the GEO database. Strawberry Perl (version 5.32.0; see text footnote 2) was used to extract gene expression data from the GSE35640 datasets and construct a data matrix for further analysis.

### Statistical Analysis

All analyses were performed with R version 4.0.4, 64-bit^6^ and its appropriate packages. We used the Kaplan-Meier survival plotter and the log-rank test to explore prognostic values and to compare the survival of patients between subgroups from each dataset. For comparison between two groups, the Wilcoxon test was used as a non-parametric method. For comparisons between more than two groups, the Kruskal-Wallis test was used as a non-parametric method. The correlation of ICI score subgroups and somatic mutation frequency was analyzed using the chi-square test. The Spearman correlation analysis was used to analyze correlation coefficients. For all statistical analyses, two-tailed *p* < 0.05 was considered statistically significant.

## Results

### Characterization of the Immune Cell Landscape in UVM

**FIGURE 1:**
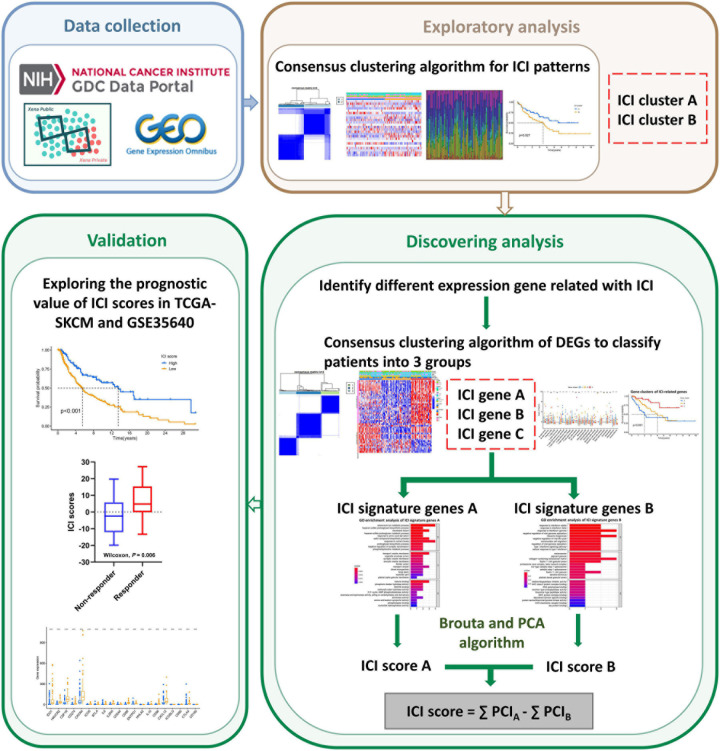
Flow chart of the study.

**FIGURE 2:**
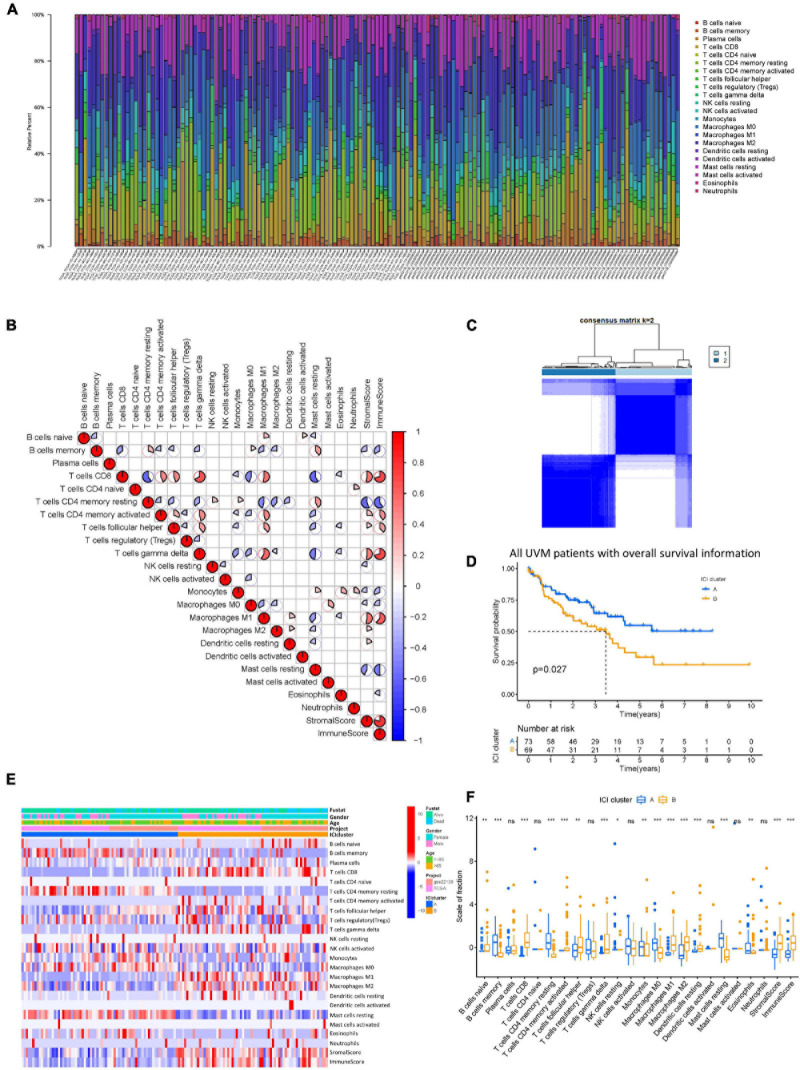
The landscape of ICI in uveal melanoma and the characteristics of ICI subtypes. **(A)** The bar plot shows the proportion of 22 infiltrating immune cells based on the CIBERSORT algorithm in the TCGA and GEO datasets. **(B)** The correlation matrix of all 22 infiltrating immune cells. The fraction of immune cells were positively related and are represented in red, whereas others were negatively related and are represented in blue. *p* < 0.05 was the cut-off. **(C)** Consensus matrix of all UVM cohorts for *k* = 2. **(D)** Kaplan–Meier curves for OS of all UVM patients with ICI classes. Log-rank test shows overall *p* = 0.027. **(E)** Unsupervised clustering and hierarchical clustering of ICI in UVM cohorts. Survival status, genders, ages, project, and ICI cluster group are shown as patient annotations. **(F)** The fraction of tumor-infiltrating immune cells in ICI clusters. We also plotted the immune and stromal scores of three gene clusters. ns, not significant; *p < 0.05; **p < 0.01; and ***p < 0.001. ICI, immune cell infiltration; UVM, uveal melanoma; OS, overall survival.

**FIGURE 3:**
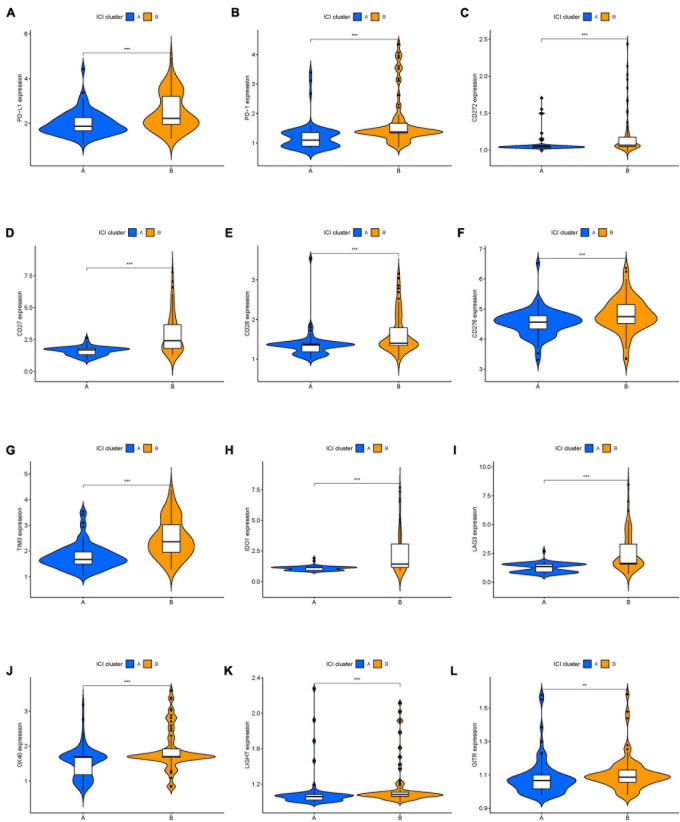
The expression levels of immune checkpoint-related genes in different ICI clusters of UVM. **(A)** PD-L1. **(B)** PD-1. **(C)** CD272. **(D)** CD27. **(E)** CD28. **(F)** CD276. **(G)** TIM3. **(H)** IDO1. **(I)** LAG3. **(J)** OX40. **(K)** LIGHT. **(L)** GITR. *p < 0.05; **p < 0.01; and ***p < 0.001. UVM, uveal melanoma.

The workflow of our study is shown in Figure 1. To analyze the landscape of ICI patterns and TME signatures in UVM, we used the CIBERSORT and ESTIMATE algorithms to quantify the profiles of immune cells in the TME of UVM tumor samples. As shown in Figure 2A, we used CIBERSORT to identify the proportion of 22 types of immune cells in the TCGA-UVM and GSE22138 datasets. Differential correlation patterns among the landscape of immune cells in the TME were visualized as a heatmap (Figure 2B). To further clarify the intrinsic biological differences in TME cell-infiltrating patterns, we used the “ConsensusClusterPlus” R package to select an optimal cluster number. We identified a significant reduction in delta area for *k* = 2 subtype clustering (Figure 2C) and delta area entered into plateau when *k* > 2 (Supplementary Figure 1). We confirmed that two independent ICI subtypes were sufficient for illustrating the variance between the UVM datasets. Two main ICI clusters revealed by our data showed significant differences in survival (log-rank test, *p* = 0.027, Figure 2D). To further characterize the intrinsic differences between immune cells and clinical data among these ICI subtypes, we used a heatmap to depict ICI phenotypes and clinical differences in the TCGA-UVM and GSE22138 cohorts (Figure 2E). Cluster-A was characterized by elevated infiltration of memory B cells, memory resting CD4^+^ T cells, resting NK cells, monocytes, M0 macrophages, resting mast cells, and eosinophils. Cluster-B was marked by infiltration of naive B cells, CD8^+^ T cells, memory activated CD4^+^ T cells, follicular helper T cells, gamma delta T cells, M1 macrophages, M2 macrophages, and resting dendritic cells. Additionally, Cluster-B was associated with a higher stromal score and immune score (Figure 2F). We also analyzed 12 immune checkpoint genes that were assessed in each ICI subtype. The Kruskal-Wallis test was used to measure the difference in expression levels of immune checkpoint-related genes in the ICI clusters. Our results show significantly increased expression levels among these immune checkpoint-related genes in the ICI cluster B group, suggesting a distinct reaction of ICB application in different ICI clusters (Figures 3A–L).

### Identification of Immune-Related Gene Subtypes in UVM

**FIGURE 4:**
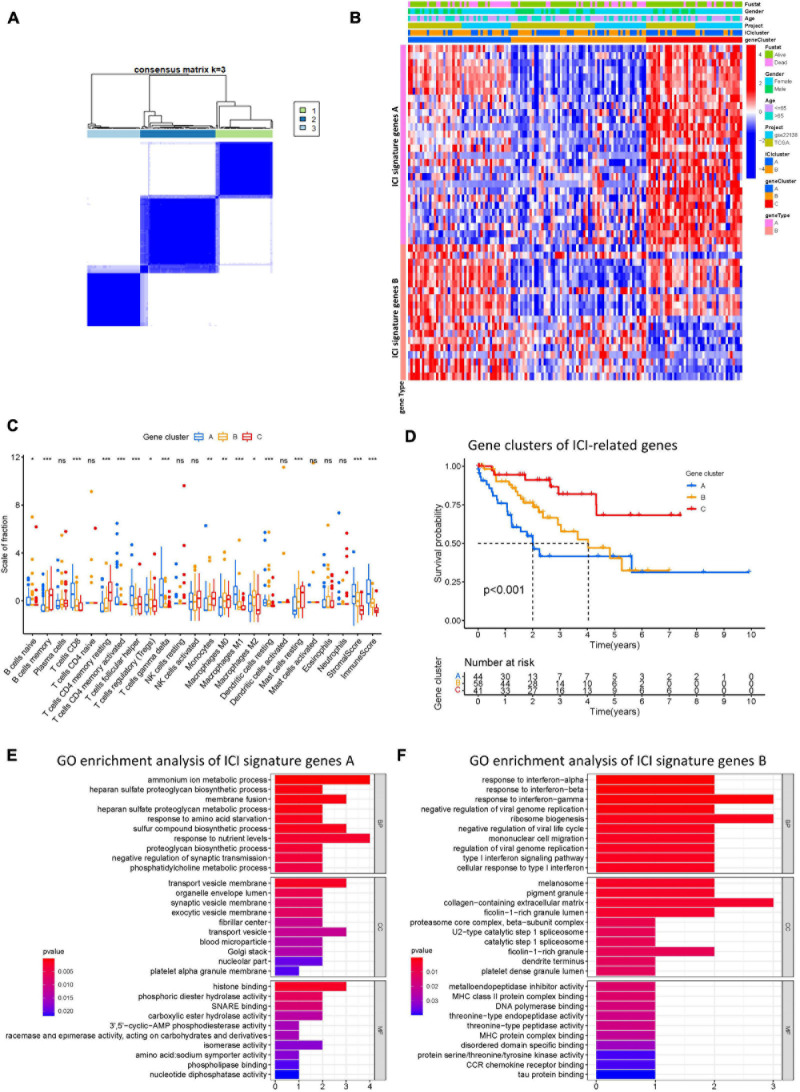
Construction of ICI gene subtypes and functional annotation. **(A)** Consensus matrix of all UVM cohorts for *k* = 3. **(B)** Unsupervised clustering and hierarchical clustering of DEGs among UVM cohorts to classify patients into three groups: Gene clusters A–C. Survival status, genders, ages, project, and ICI cluster group are shown as patient annotations. **(C)** The fraction of tumor-infiltrating immune cells in ICI gene clusters. We also plotted the immune and stromal scores of three gene clusters. **(D)** Kaplan–Meier curves for the three gene clusters of UVM patients. Log-rank test shows overall *p* < 0.001. **(E,F)** GO enrichment analysis of the two ICI relevant signature genes: ICI signature gene A **(E)** and B **(F)**. ns, not significant; *p < 0.05; **p < 0.01; and ***p < 0.001. ICI, immune cell infiltration; UVM, uveal melanoma; OS, overall survival; GO, gene ontology.

**FIGURE 5:**
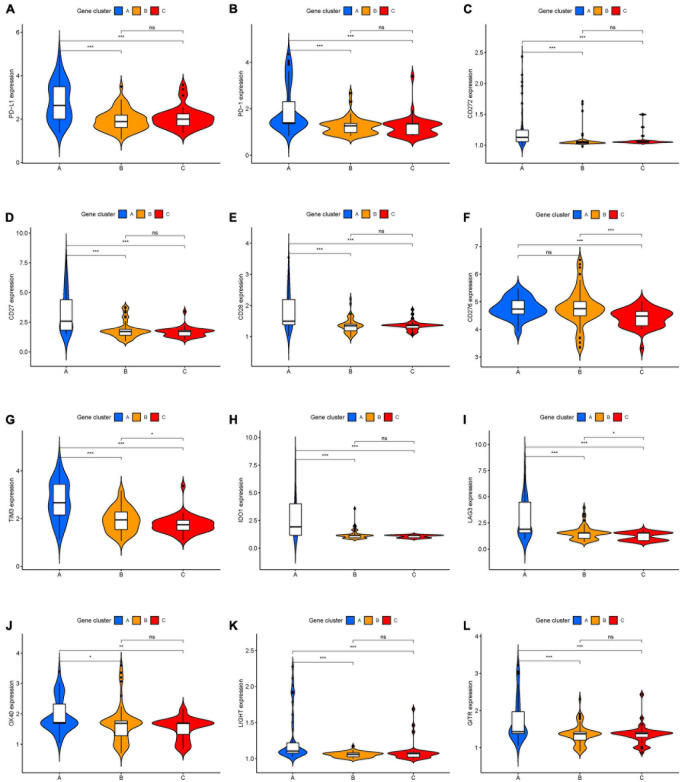
The expression levels of immune checkpoint-related genes in different ICI genes subtypes of UVM. **(A)** PD-L1. **(B)** PD-1. **(C)** CD272. **(D)** CD27. **(E)** CD28. **(F)** CD276. **(G)** TIM3. **(H)** IDO1. **(I)** LAG3. **(J)** OX40. **(K)** LIGHT. **(L)** GITR. ns, not significant; *p < 0.05; **p < 0.01; and ***p < 0.001. UVM, uveal melanoma.

To identify genes associated with our ICI clusters, we performed differential gene analyses to detect DEGs among these ICI subtypes by using “Limma” R packages. We performed unsupervised clustering of DEGs to select the optimal gene cluster number, which resulted from the previous analysis. Next, we sought to use “Boruta” algorithms to perform dimension reduction in the ICI-related genes to reduce redundant genes and extract phenotype signatures. The unsupervised hierarchical cluster analysis classified the TCGA-UVM and GSE22138 cohorts into three gene clusters termed gene clusters A–C (Figure 4A and Supplementary Figure 2). Moreover, differential correlation patterns among the ICI gene clusters were visualized as a heatmap (Figure 4B). As shown in Figure 4C, the boxplot indicates significant differences in tumor-related ICI and the stromal score and immune score in the three ICI gene clusters. Among the three main ICI gene clusters, we found that gene cluster A was associated with a significant level of CD8 + T cells, memory activated CD4 + T cells, follicular helper T cells, gamma delta T cells, M1 macrophages, resting dendritic cells, stromal score, and immune score. Additionally, we also analyzed the expression of immune checkpoint-related genes in each ICI gene cluster. These genes were elevated in the ICI gene A cluster group, suggesting a distinct reaction of ICB application in different ICI clusters (Figures 5A–L). UVM patients in the cluster A might exhibit a better response to immune checkpoint inhibitors, such as cytotoxic T lymphocyte-associated antigen-4 (CTLA4) and programmed cell death protein 1 (PD1) inhibiting reagents.

Prognostic analysis of the ICI gene clusters was conducted using the R packages “survival” and “survminer.” Using Kaplan-Meier survival analysis, we found that ICI gene clusters B and C correlated with relatively good prognosis in UVM, and ICI gene cluster A were associated with poorer prognosis (log-rank test, *p* < 0.001; Figure 4D). DEG expression levels that were either positively or negatively correlated with the ICI gene cluster signature were then classified as two subgroups: ICI gene signatures A and B. By using the R package “clusterProfiler,” we performed GO enrichment analysis of the ICI gene signatures. We found that ICI gene signature A was closely linked with a variety of terms, including “ammonium ion metabolic process” in the BP category, “transport vesicle membrane” in the CC category, and “histone binding” in the MF category (Figure 4E). ICI gene signature B showed enrichment of “response to interferon-gamma” in the BP category, “collagen-containing extracellular matrix” in the CC category, and “metalloendopeptidase inhibitor activity” in the MF category (Figure 4F).

### Calculation of ICI Scores

**FIGURE 6:**
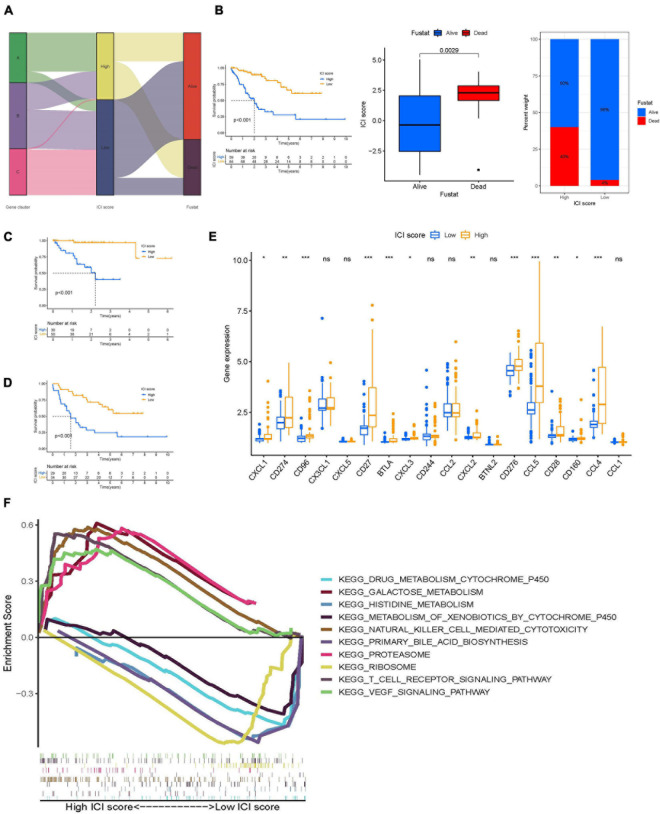
Construction of ICI scores. **(A)** Alluvial diagram of ICI gene clusters in groups with different ICI clusters, ICI scores, and survival outcomes. **(B)** Kaplan–Meier curves for the high- and low-ICI scores of UVM patients in TCGA-UVM and GSE22138 cohorts. Log-rank test shows overall *p* < 0.001. Distribution of ICI scores in different survival status in UVM (*p* = 0.029). Rate of in high- and low-ICI score subgroups in UVM. **(C,D)** Kaplan–Meier curves for the high- and low-ICI scores of UVM patients in TCGA-UVM **(C)** and GSE22138 cohorts **(D)**. Log-rank test shows overall *p* < 0.001. **(E)** The expression level of immunoinhibitory signatures genes (BTLA, CD160, CD244, CD274, and CD96), immunostimulatory signatures genes (BTNL2, C10orf54, CD27, CD276, and CD28), and chemokine signatures genes (CCL1, CCL2, CCL3, CCL4, CCL5, CX3CL1, CXCL1, CXCL2, CXCL3, and CXCL5) in the high- and low-ICI score subgroup. ns, not significant; *p < 0.05; **p < 0.01; and ***p < 0.001. **(F)** Enriched gene sets annotated by KEGG collection between the high- and low-ICI score subgroup. ICI, immune cell infiltration; UVM, uveal melanoma; OS, overall survival; KEGG, Kyoto encyclopedia of genes and genomes.

To discern an indicator for the ICI landscape, we defined two aggregate scores using the PCA algorithm: ICI score A from ICI gene signature A and ICI score B from ICI gene signature B. ICI scores A and ICI scores B were then computed as the sum of relevant individual scores. Finally, we obtained the prognostic signature score that is termed ICI score. We classified the TCGA-UVM and GSE22138 cohorts into two groups, namely high- and low-ICI scores. We visualized changes in clusters using an alluvial diagram; gene clusters B and C were linked to a low-ICI score (Figure 6A) and were associated with a better prognostic outcome in UVM cohorts (Figure 6B). Furthermore, we evaluated the prognostic implications of the ICI scores by integrating them with survival data. Using Kaplan-Meier survival analysis, we found that patients in the high-ICI score group had worse survival outcomes in the TCGA-UVM cohort (log-rank test, *p* < 0.001; Figure 6C). We also evaluated the prognostic accuracy of the ICI scores in the GSE22138 cohort (log-rank test, *p* < 0.001; Figure 6D). Furthermore, we analyzed the immune activity and chemokine profiles in the high- and low ICI score groups. To evaluate this association, we selected BTLA, CD160, CD244, CD274, and CD96 as immunoinhibitory signatures; BTNL2, C10orf54, CD27, CD276, and CD28 as immunostimulatory signatures; and CCL1, CCL2, CCL3, CCL4, CCL5, CX3CL1, CXCL1, CXCL2, CXCL3, and CXCL5 as chemokine signatures. We found that most immune checkpoint signatures and chemokines, except CX3CL1, CXCL5, CD244, CCL2, BTNL2, and CCL1, were significantly overexpressed in the high-ICI score group (Figure 6E). Additionally, multiple-GSEA was performed on ICI score gene signatures. We found that VEGF signaling and T-cell receptor signaling were significantly enriched in the high-ICI score group, whereas histidine metabolism and ribosome metabolism were significantly enriched in the low-ICI score group (Figure 6F).

### Correlation of ICI Score With Tumor Mutational Burden

**FIGURE 7:**
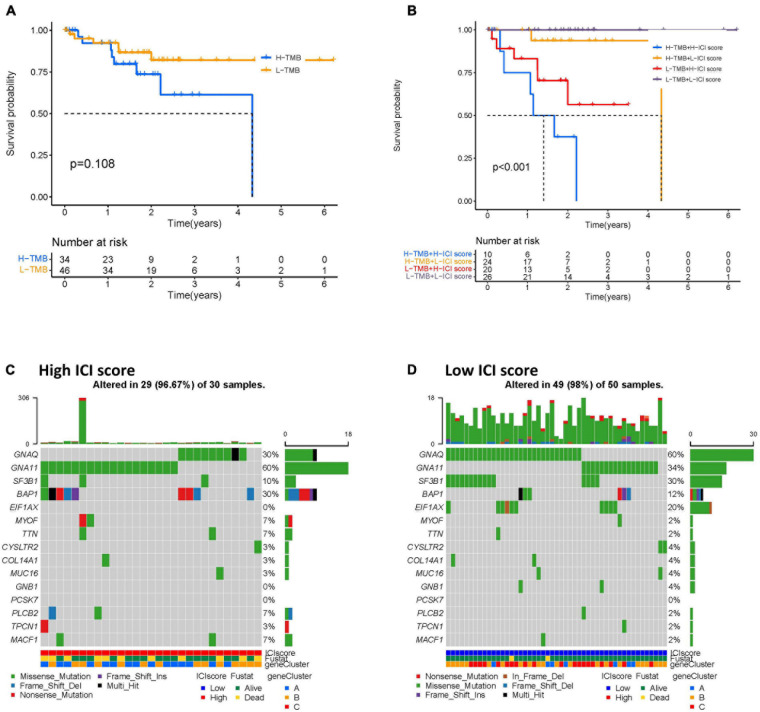
The correlation between the ICI scores and TMB. **(A)** Kaplan–Meier curves for the high- and low-TMB of UVM patients. Log-rank test shows overall *p* = 0.108. **(B)** Kaplan–Meier curves for UVM patients in TMB and ICI score subgroups. Log-rank test shows overall *p* < 0.001. **(C,D)** The oncoPrint was constructed by the high- **(C)** and low-ICI scores **(D)** of UVM patients. Individual patients are represented in each column. UVM, uveal melanoma; ICI, immune cell infiltration; TMB, tumor mutational burden.

To identify the intrinsic correlation between TMB and ICI score, we compared the TMB of high- and low-ICI score groups. We first analyzed the correlation between patient prognosis and TMB level. As shown in Figure 7A, we found no significant difference between TMB level and patient prognosis. Further correlation analyses confirmed no significant correlation between the two (Supplementary Figure 3). We next investigated the effect of ICI score and TMB level pattern in the UVM cohort. We found that patients with high-TMB and high-ICI scores had a poorer prognosis when compared to the other three subgroups (log-rank test, *p* < 0.001; Figure 7B). Furthermore, we analyzed the distribution of TMB in high- and low-ICI score groups by using the “maftools” R package. The top 15 genes with the highest mutational frequency were selected and visualized (Figures 7C,D). Taken together, the correlations between TMB level and ICI score in UVM implies that TMB might play an essential role in predicting patient outcomes and response to ICB therapy.

### Correlation of ICI Score With Gender and Age

**FIGURE 8:**
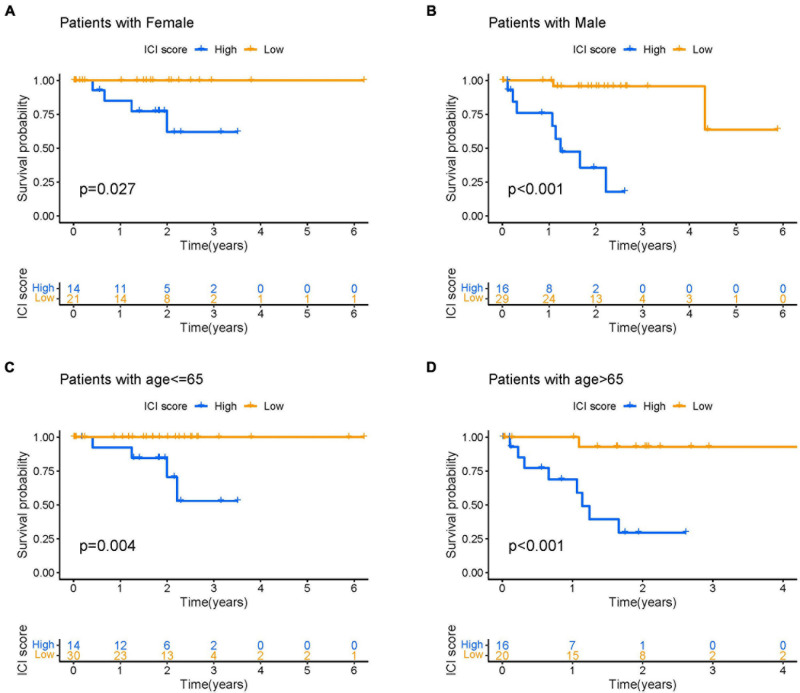
The correlation of ICI scores with patient gender and age. **(A,B)** Kaplan–Meier curves for the high- and low-ICI scores of female **(A)** and male **(B)** UVM patients in the TCGA-UVM and GSE22138 cohorts, respectively. Log-rank test shows overall *p* = 0.027 and *p* < 0.001, respectively. **(C,D)** Kaplan–Meier curves for the high- and low-ICI scores of UVM patients with age ≤ 65 **(C)** and age > 65 **(D)** in the TCGA-UVM and GSE22138 cohorts, respectively. Log-rank test shows overall *p* = 0.004 and *p* < 0.001, respectively. UVM, uveal melanoma; ICI, immune cell infiltration.

We performed a comprehensive analysis to investigate the feasibility of correlating ICI score with patient gender and age in UVM. The analysis involved evaluating prognostic implication of ICI score in different genders. Kaplan-Meier analysis showed that both male and female patients with high ICI scores had a significantly poorer prognosis than those with low ICI scores (log-rank test, *p* < 0.0001; Figures 8A,B). Next, we categorized UVM patients into two subgroups based on age (>65 and ≤65). As demonstrated in Figures 8C,D, we found that patients with high ICI scores showed a poorer prognosis than those with low ICI scores regardless of age.

### ICI Score Predicts Immunotherapeutic Benefit

**FIGURE 9:**
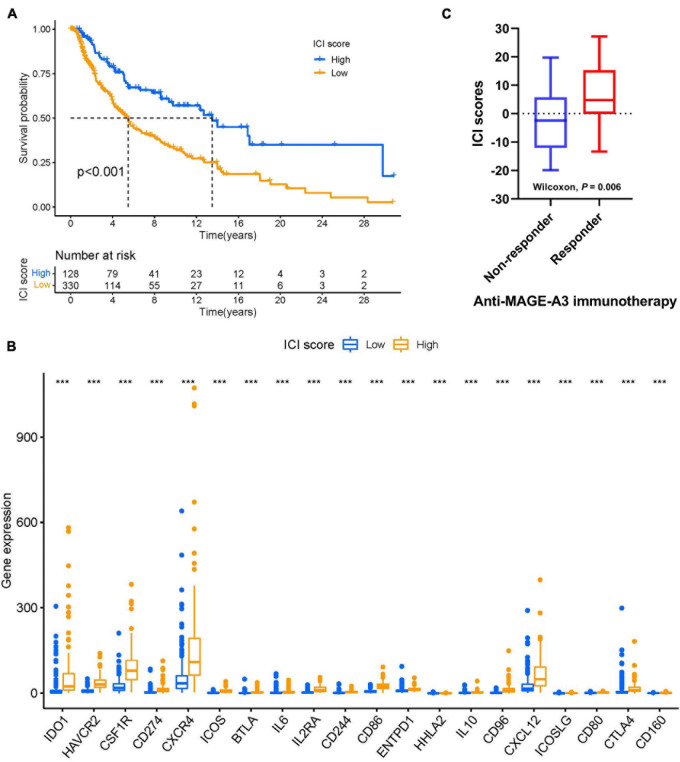
ICI scores predict immunotherapeutic benefit. **(A)** Kaplan–Meier curves for the high- and low-ICI scores of skin melanoma patients in TCGA-SKCM. Log-rank test shows overall *P* < 0.001. **(B)** The expression levels of immune checkpoint-related genes in different ICI score subtypes of SKCM. ***P < 0.001. **(C)** ICI scores in responders versus non-responders in the anti-MAGE-A3 immunotherapy cohort (Wilcoxon *p* = 0.06). Medians, interquartile ranges, and minimum/maximum are shown in the boxplot. ***p < 0.001. SKCM, skin cutaneous melanoma; ICI, immune cell infiltration.

We next explored the possibility of using ICI score to predict immune-checkpoint therapy response. Two transcriptomic data sets from patients treated with various types of immunotherapies from the TCGA-SKCM cohort and patients with advanced melanoma treated with MAGE-3 antigen-based immunotherapy were downloaded and analyzed to determine the predictive value of ICI score. As demonstrated in Figure 9A, we found that patients with low ICI scores showed worse survival outcomes than those with high ICI scores in the TCGA-SKCM cohort (log-rank test, *p* < 0.001). We also analyzed immunoinhibitory, immunostimulatory, and immune-checkpoint-relevant genes in each group in the TCGA-SKCM cohort. We found that BTLA, CD160, CD244, CD274, CD96, CSF1R, CTLA4, HAVCR2, IDO1, IL10, CD80, CD86, CXCL12, CXCR4, ENTPD1, HHLA2, ICOS, ICOSLG, IL2RA, and IL6 were significantly overexpressed in the high-ICI score group, as demonstrated in Figure 9B. Next, we sought to validate the predictive value of ICI score in SKCM patients treated with anti-MAGE-A3 antigen-specific cancer immunotherapy. Patients with high ICI scores were more likely to respond to immune-checkpoint therapy (Figure 9C). Collectively, our results suggest that ICI scores correlate well with response to immunotherapy.

## Discussion

Despite the remarkable progress of ICB therapy in metastatic melanoma, UVM remains immunotherapy-resistant (Wierenga et al., 2019). Over the past several years, infiltration of immune cells has been identified as a predictive and prognostic biomarker in melanoma (Nakamura and Okuyama, 2016; Leonardi et al., 2020). Recent clinical trials reported that the disease control rate among UVM patients is 71% for tebentafusp (also known as IMCgp100) therapy. Its mechanism is based on the activation of T cells, resulting in tumor cell lysis. Control rates vary for other therapies, from 64% for combined immunotherapy (CTLA-4 plus anti-PD-1) to 43% for tumor-infiltrating lymphocyte therapy. In addition, patients with metastatic UVM have been successfully treated using checkpoint inhibition, exhibiting prolonged progression-free survival and overall survival rates, or even complete remission in some cases (Falzone et al., 2018; Christofi et al., 2019; Schank and Hassel, 2019). Yet the treatment of metastatic UVM remains challenging, and further therapeutic development is needed (Kaštelan et al., 2020). Evolving a better understanding of the phenotype and function of ICI in the context of ICB therapy and other factors within the TME is crucial to identifying patients most likely to respond. Our study establishes a methodology to characterize ICI patterns in UVM using TCGA and GEO databases. Our findings indicate that ICI scores provide a robust prognostic biomarker in patients with UVM and predict response to ICB therapy. The outcomes of our study demonstrate (i) estimation of ICI patterns in patients with UVM; (ii) correlation between high ICI scores and poor prognosis; (iii) correlation between somatic alterations and prognosis; (iv) association of ICI score with the response to ICB therapy.

A myriad of evidence has demonstrated that tumor-related immune cells within the UVM microenvironment promote immunosuppression and tumor immune escape. For instance, TILs, such as CD4 + T cells and CD8 + T cells, play an essential role in tumor dissemination, relapse, metastasis, and therapeutic response to immunotherapy (Vassilakopoulou et al., 2016; Jiang et al., 2018; Zeng et al., 2018). TIL grade may be considered an independent predictor of melanoma-specific survival and recurrence-free survival (Gata et al., 2017). Thus, TILs have been supported as a therapeutic target for predicting and optimizing the response to immunotherapy in melanoma (Egger et al., 2016). A high density of CD8^+^ T cells has been shown to predict poor 5-year overall survival (Wang et al., 2020). In our analysis, Cluster B was marked by high densities of CD8^+^ T cells, CD4^+^ memory-activated T cells, follicular helper cells, gamma delta T cells, M1 macrophages, M2 macrophages, and dendritic cells, which were correlated with poor prognosis. This emphasizes the fact that pre-existing immune responses might affect the response to immunotherapy. In addition, a series of immunosuppressive cytokines and chemokines might affect anti-tumor responses, including transforming growth factor-beta (TGF-β), chemokines, and prostaglandin E2 (PGE2) (Gorelik and Flavell, 2001; Nicolaou et al., 2004). Blockade of TGF-β signaling in murine cancer models was found to promote tumor-specific immunity (Gorelik and Flavell, 2001). Several chemokines may act as a dual role with regards to their pro- and anti-cancer activities in melanoma tumors (Harlin et al., 2009; Bagheri et al., 2020).

We also investigated the relationship between ICI and the pattern of immune-related gene expression. We identified immune-related genes and described three novel ICI gene clusters. Our results show that ICI gene cluster A was associated with poor prognosis. We found that the TIL of gene cluster A was composed of CD8^+^ T cells, T follicular helper cells, gamma delta T cells, and M1 macrophages. Interestingly, gene cluster A exhibited higher stromal and immune scores. In accordance with the previous study, high immune and stromal scores were associated with poor prognosis (García-Mulero et al., 2021). Through multiple-GSEA analysis, we found that VEGF signaling (Ishikawa et al., 2021) and T-cell receptor signaling (Yang et al., 2021) was significantly enriched in the high-ICI score subgroup.

In our study, somatic variants and chromosomal aberrations were correlated with clinical outcomes. TMB (non-synonymous variants) is significantly associated with the efficacy of immunotherapy (Goodman et al., 2017). In contrast to cutaneous melanoma, UM is considered an “immune-cold” tumor due to its low TMB and its distinct TME (Galon and Bruni, 2019). Analysis of the mutation annotation files of the UVM cohort revealed that the alteration frequency of *GNAQ*, *EIF1AX*, and *GNA11* was significantly different between high- and low-ICI score groups (Supplementary Table 2). Mutation of *GNAQ* or *GNA11* occurs in more than 90% of UVMs and plays a crucial role in the activation of oncogenic pathways, including MAPK and YAP (Van Raamsdonk et al., 2010). A better understanding of UVM mutational status may help clinicians to select the most effective immunotherapy. For instance, recent studies suggest that metastatic UVM may be responsive to MEK inhibitors in *GNAQ11*-driven melanoma (Truong et al., 2020). We also analyzed the relationship between ICI score and TMB. However, we did not observe an independent predictive advantage for TMB. Combining TMB and ICI scores demonstrated a synergistic effect in predicting prognosis in UM patients. Our study shows that the prognostic value of ICI score is independent of TMB in UVM patients; however, further experimentation is needed to confirm this result.

Cancer immunotherapy manipulates the host’s immune system to recognize and attack cancer cells. It has shown extraordinary progress in patients with cutaneous melanoma, including PD-L1 and anti-cytotoxic T-lymphocyte antigen-4 (CTLA-4) checkpoint inhibitors (Boutros et al., 2016). However, these results have not been reproduced in UM (Rossi et al., 2019), mainly because UM is different from CM at the genetic and molecular level, necessitating the use of targeted therapy (Algazi et al., 2016). Here, we demonstrate the predictive value of ICI score for advanced melanoma patients treated with anti-MAGE-A3 blocker (Ulloa-Montoya et al., 2013). We observed that ICI scores were significantly higher in melanoma patients responding to checkpoint blockade therapy. This suggests that single-agent ICB therapy may be effective in patients with high ICI scores.

This study has several limitations. All our results were theoretical and based on sequencing data. Thus, large-scale clinical investigation is needed to validate our conclusions. The TCGA-UVM cohorts and GSE22138 cohort are the largest UVM cohorts available (80 samples and 63 samples, respectively) and are recognized by most institutions. However, this sample size is not representative of the size of the population with melanoma. Due to limited data from the melanoma cohort, the ICI signatures identified in this study require confirmation both *in vivo* and *in vitro*.

In summary, our study assessed the ICI landscape in UVM and highlights the association between ICI and tumor heterogeneity. It represents a comprehensive analysis of the TCGA and GEO databases. Finally, it may help clinicians develop novel, potent ICB therapies.

## Data Availability Statement

The original contributions presented in the study are included in the article/Supplementary Material, further inquiries can be directed to the corresponding author.

## Author Contributions

HZ and YC contributed to conception, design, and data acquisition and interpretation, and drafted and critically revised the manuscript. PS contributed to data acquisition and interpretation, and critically revised the manuscript. LG contributed to design and critically revised the manuscript. All authors gave their final approval and agreed to be accountable for all aspects of the work.

## Conflict of Interest

The authors declare that the research was conducted in the absence of any commercial or financial relationships that could be construed as a potential conflict of interest.

## Publisher’s Note

All claims expressed in this article are solely those of the authors and do not necessarily represent those of their affiliated organizations, or those of the publisher, the editors and the reviewers. Any product that may be evaluated in this article, or claim that may be made by its manufacturer, is not guaranteed or endorsed by the publisher.

## Footnotes

1
https://xenabrowser.net/

2
http://strawberryperl.com

3
http://www.gsea-msigdb.org/gsea/msigdb/index.jsp

4
http://www.gsea-msigdb.org/

5
https://portal.gdc.cancer.gov/

6
https://www.r-project.org/

## Supplementary Material

Supplementary Figure 1 Consensus matrix of all UVM cohorts for each k, except for *k* = 2, and displaying the clustering stability using 1,000 iterations of hierarchical clustering. **(A)** *k* = 3. **(B)** *k* = 4. **(C)** *k* = 5. **(D)** *k* = 6. **(E)** *k* = 7. **(F)** *k* = 8. **(G)** *k* = 9. **(H)** Consensus clustering CDF for *k=2* to *k=9*. **(I)** Delta area plot showing the relative change in area under the CDF curve from *k=2* to *k=9*.

Supplementary Figure 2 Consensus matrix of all UVM cohorts for each k, except for *k* = 3, displaying the clustering stability using 1,000 iterations of hierarchical clustering. **(A)** *k=2*. **(B)** *k* = 4. **(C)** *k* = 5. **(D)** *k* = 6. **(E)** *k* = 7. **(F)** *k* = 8. **(G)** *k* = 9. **(H)** Consensus clustering CDF for *k=2* to *k=9*. **(I)** Delta area showing the relative change in area under the CDF curve from *k=2* to *k=9*. **(J)** Tracking plot for *k=2* to *k=9*.

Supplementary Figure 3 The correlation between the ICI scores and somatic alterations. **(A)** TMB difference levels in the high- and low-ICI score subgroups (Wilcoxon *p* = 0.63). **(B)** Scatter plot showing the correlation between high- and low-ICI score subgroups and TMB (*R* = −0.028, *p* = 0.81). ICI, immune cell infiltration; TMB, tumor mutational burden.

Supplementary Table 1 The clinical and pathological characteristics of uveal melanoma patients in the TCGA-UVM and GSE22138 cohorts.

Supplementary Table 2 The correlation between the ICI scores and TMB.
