# Supplementary material for: Identification of Immune Cell Infiltration Landscape and Their Prognostic Significance in Uveal Melanoma
Source: Front Cell Dev Biol. 2021 Aug 26;9:713569. doi: 10.3389/fcell.2021.713569 (PMC8427429; doi:10.3389/fcell.2021.713569)
Supplement: Supplementary Figure 1 — Consensus matrix of all UVM cohorts for each k, except for k = 2, and displaying the clustering stability using 1,000 iterations of hierarchical clustering. (A) k = 3. (B) k = 4. (C) k = 5. (D) k = 6. (E) k = 7. (F) k = 8. (G) k = 9. (H) Consensus clustering CDF for k=2 to k=9. (I) Delta area plot showing the relative change in area under the CDF curve from k=2 to k=9. [file Data_Sheet_1.docx]

Supplementary Material

# Supplementary Figures and Tables

## Supplementary Figures


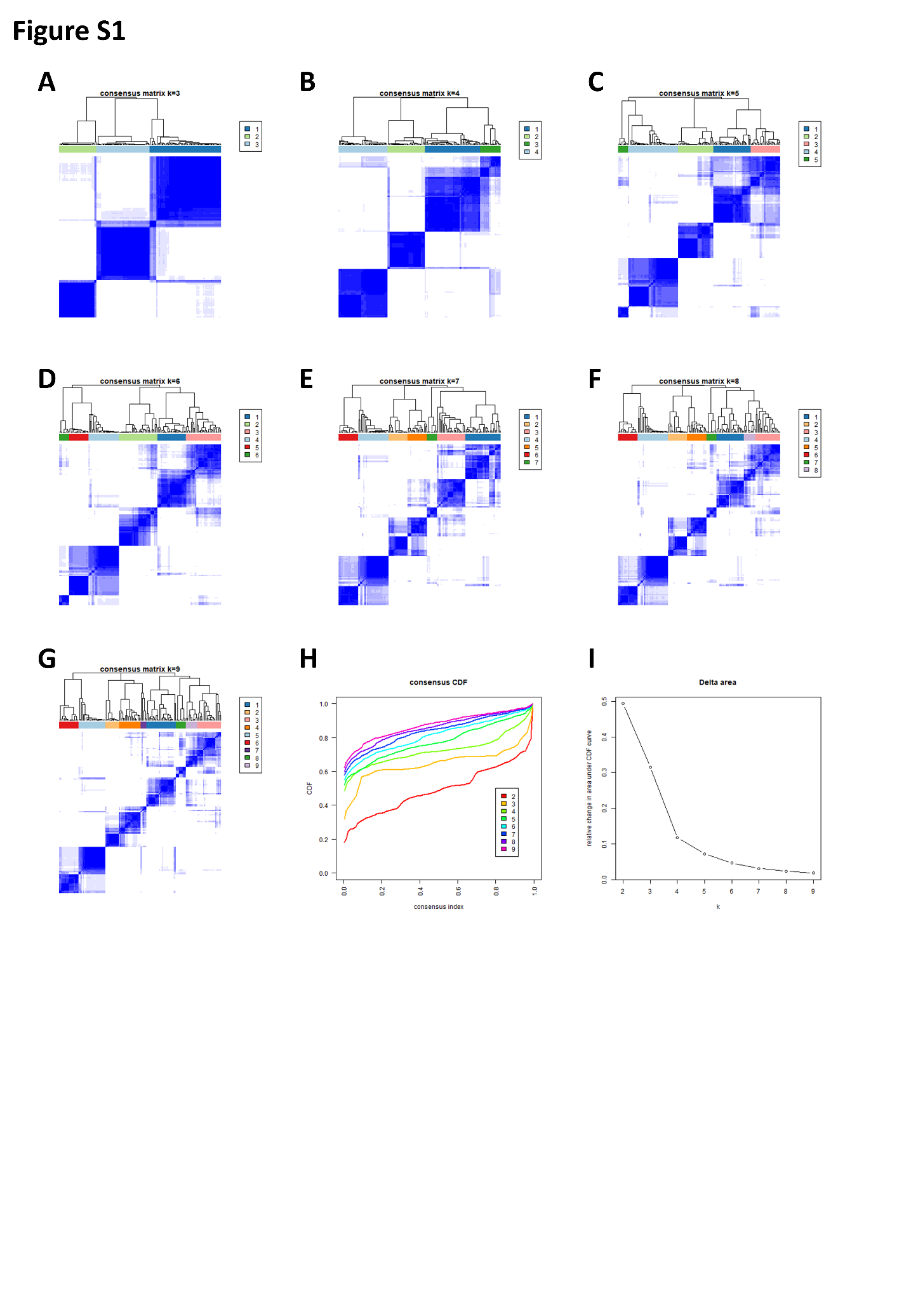


**Supplemental Figure S1.** Consensus matrix of all UVM cohorts for each k, except for k = 2, and displaying the clustering stability using 1000 iterations of hierarchical clustering.

**
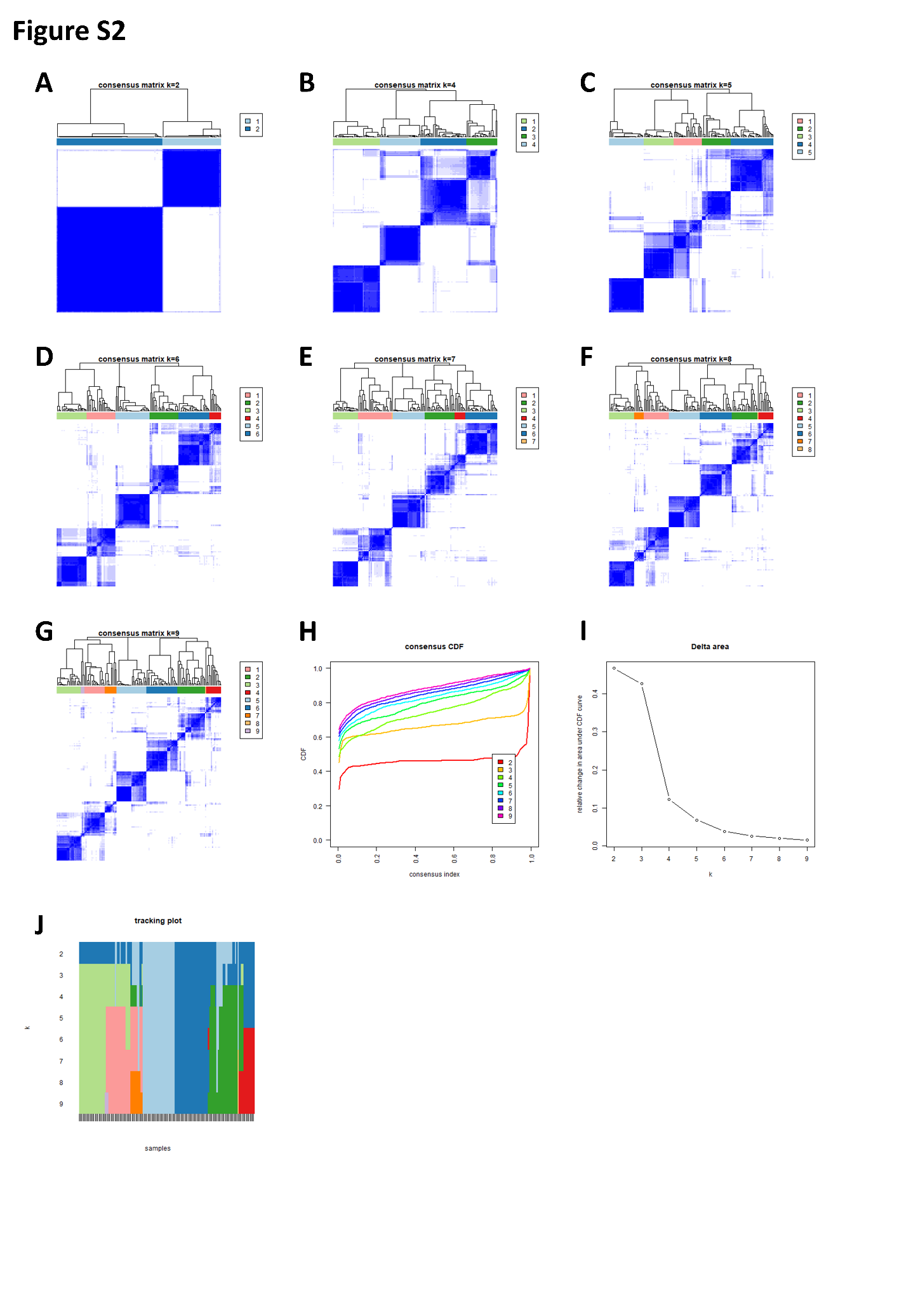
**

**Supplemental Figure S2.** Consensus matrix of all UVM cohorts for each k, except for k = 3, displaying the clustering stability using 1000 iterations of hierarchical clustering.

**
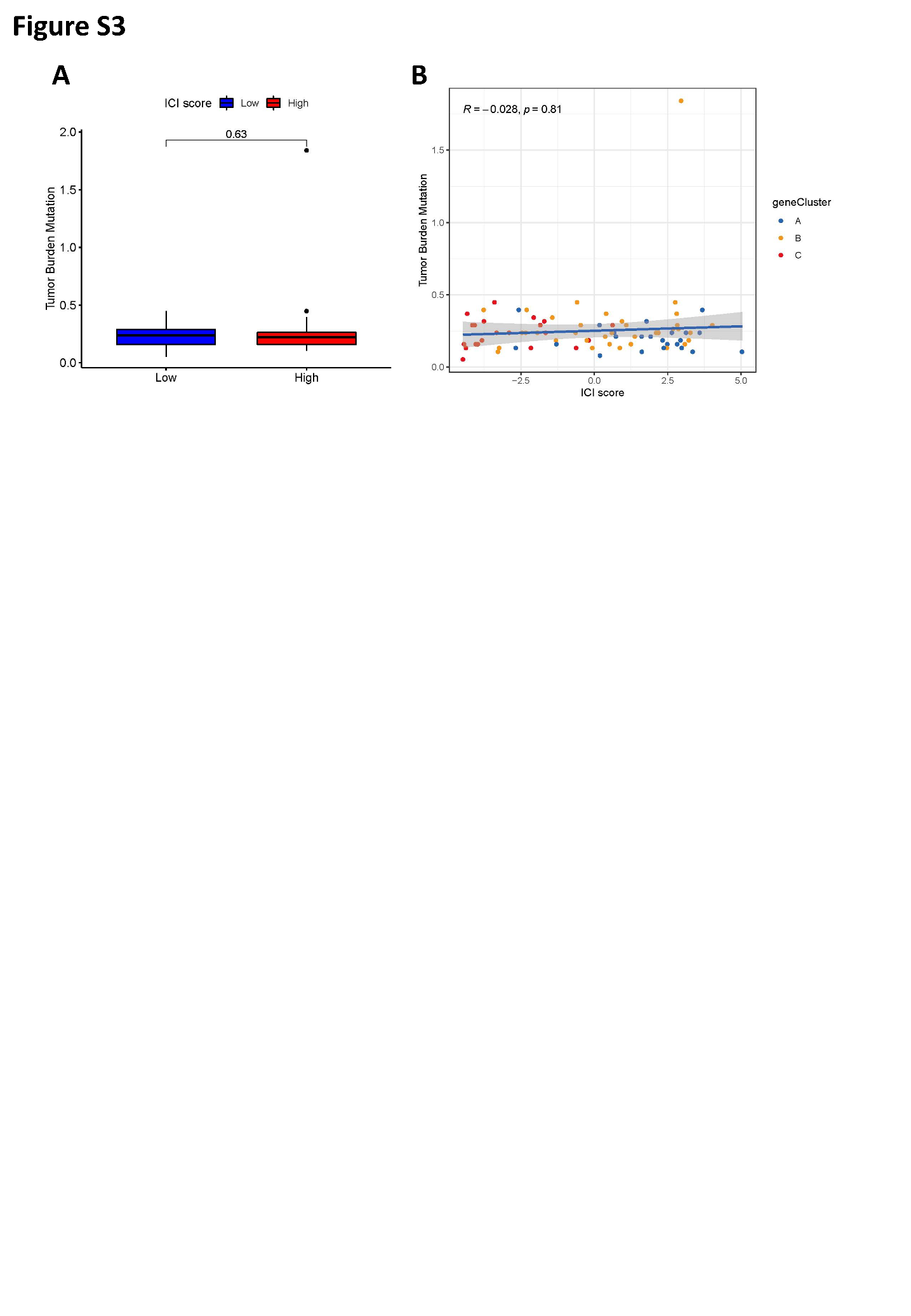
**

**Supplemental Figure S3.** The correlation between the ICI scores and somatic alterations. (A) TMB difference levels in the high- and low-ICI scores subgroups (Wilcoxon p = 0.63). (B) The scatter plot for showing the correlation between high- and low-ICI scores subgroups and TMB (R = - 0.028, p = 0.81). ICI, immune cell infiltration; TMB, tumor mutation burden.

**Supplemental Table S1.** The clinical and pathological characteristics of uveal melanoma patients in the TCGA-UVM and GSE22138 cohorts.

| **Characteristics** | **Training Cohort**  **(TCGA-UVM, 80 Cases)** | **Validation Cohort**  **(GSE22138, 63 Cases)** |
| --- | --- | --- |
| Age at diagnosis, years |  |  |
| <65 | 45(56.25%) | 36(57.14%) |
| ≥65 | 35(43.75%) | 27(42.86%) |
| gender |  |  |
| female | 35(43.75%) | 24(38.1%) |
| male | 45(56.25%) | 39(61.9%) |
| T classification |  |  |
| T1 | 0 | NA |
| T2 | 4(5%) | NA |
| T3 | 36(45%) | NA |
| T4 | 38(47.5%) | NA |
| unknown | 2(2.5%) | NA |
| M classification |  |  |
| M0 | 73(91.25%) | 28(44.44%) |
| M1 | 3(3.75%) | 35(55.56%) |
| unknown | 4(5%) | 0 |
| N classification |  |  |
| N0 | 76 (95.00%) | NA |
| N1 | 0 (0.00%) | NA |
| unknown | 4 (5.00%) | NA |
| tumor stage |  |  |
| stage I | 0 | NA |
| stage II | 36(45%) | NA |
| stage III | 40(50%) | NA |
| stage IV | 4(5%) | NA |
| tumor eye side |  |  |
| left | NA | 33(52.38%) |
| right | NA | 30(47.62%) |
| tumor cell type |  |  |
| epithelioid | NA | 21(33.33%) |
| mixed | NA | 23(36.51%) |
| unknown | NA | 19(30.16%) |
| tissue or organ of origin diagnosis |  |  |
| choroid | 67(83.75%) | NA |
| ciliary body | 5(6.25%) | NA |
| overlapping lesion of eye and adnexa | 8(10%) | NA |

TCGA: The Cancer Genome Atlas; UVM: Uveal Melanoma; NA: data not available.

**Supplemental Table S2.** The correlation between the ICI scores and TMB.

| **Gene** | **H-wild** | **H-mutation** | **L-wild** | **L-mutation** | ***p*-value** |
| --- | --- | --- | --- | --- | --- |
| GNAQ | 21(70%) | 9(30%) | 20(40%) | 30(60%) | 0.017891 |
| EIF1AX | 30(100%) | 0(0%) | 41(82%) | 9(18%) | 0.035619 |
| GNA11 | 12(40%) | 18(60%) | 33(66%) | 17(34%) | 0.04168 |
